# The Molecular Structure and Self-Assembly Behavior of Reductive Amination of Oxidized Alginate Derivative for Hydrophobic Drug Delivery

**DOI:** 10.3390/molecules26195821

**Published:** 2021-09-25

**Authors:** Xiuqiong Chen, Qingmei Zhu, Zhengyue Li, Huiqiong Yan, Qiang Lin

**Affiliations:** 1 Key Laboratory of Tropical Medicinal Resource Chemistry of Ministry of Education, College of Chemistry and Chemical Engineering, Hainan Normal University, Haikou 571158, China; chenxiuqiongedu@163.com; 2 Key Laboratory of Natural Polymer Functional Material of Haikou City, College of Chemistry and Chemical Engineering, Hainan Normal University, Haikou 571158, China; zhuqm03@163.com (Q.Z.); zhengyedu@163.com (Z.L.); 3 Key Laboratory of Water Pollution Treatment & Resource Reuse of Hainan Province, College of Chemistry and Chemical Engineering, Hainan Normal University, Haikou 571158, China

**Keywords:** reductive amination of oxidized alginate derivative, systematic characterization, molecular structure, self-assembly behavior, hydrophobic drug delivery

## Abstract

On account of the rigid structure of alginate chains, the oxidation-reductive amination reaction was performed to synthesize the reductive amination of oxidized alginate derivative (RAOA) that was systematically characterized for the development of pharmaceutical formulations. The molecular structure and self-assembly behavior of the resultant RAOA was evaluated by an FT-IR spectrometer, a ^1^H NMR spectrometer, X-ray diffraction (XRD), thermal gravimetric analysis (TGA), a fluorescence spectrophotometer, rheology, a transmission electron microscope (TEM) and dynamic light scattering (DLS). In addition, the loading and in vitro release of ibuprofen for the RAOA microcapsules prepared by the high-speed shearing method, and the cytotoxicity of the RAOA microcapsules against the murine macrophage RAW264.7 cell were also studied. The experimental results indicated that the hydrophobic octylamine was successfully grafted onto the alginate backbone through the oxidation-reductive amination reaction, which destroyed the intramolecular hydrogen bond of the raw sodium alginate (SA), thereby enhancing its molecular flexibility to achieve the self-assembly performance of RAOA. Consequently, the synthesized RAOA displayed good amphiphilic properties with a critical aggregation concentration (CAC) of 0.43 g/L in NaCl solution, which was significantly lower than that of SA, and formed regular self-assembled micelles with an average hydrodynamic diameter of 277 nm (PDI = 0.19) and a zeta potential of about −69.8 mV. Meanwhile, the drug-loaded RAOA microcapsules had a relatively high encapsulation efficiency (EE) of 87.6 % and good sustained-release properties in comparison to the drug-loaded SA aggregates, indicating the good affinity of RAOA to hydrophobic ibuprofen. The swelling and degradation of RAOA microcapsules and the diffusion of the loaded drug jointly controlled the release rate of ibuprofen. Moreover, it also displayed low cytotoxicity against the RAW264.7 cell, similar to the SA aggregates. In view of the excellent advantages of RAOA, it is expected to become the ideal candidate for hydrophobic drug delivery in the biomedical field.

## 1. Introduction

Alginate, mainly originated from seaweeds, is a kind of natural anionic heteropolysaccharide. It is composed of α-L-guluronic acid (G) and β-d-mannuronic acid (M) with random (1–4) glycosidic bonds [1,2,3]. As a result of their excellent advantages, such as non-toxicity, immunogenicity, low cost, good biodegradation and biocompatibility, alginate and its derivatives can be used in food, cosmetics, drug delivery vehicles, tissue engineering scaffolds, and wound dressings [4,5,6,7]. In particular, alginate can easily achieve ionic cross-linking with the use of divalent cations, usually calcium, thus forming a hydrogel with excellent encapsulation and drug loading performance [8,9] which makes it a good candidate as a drug carrier. In spite of its merits, alginate still presents numerous drawbacks that restrict its practical application in drug delivery. Raw alginate is very hydrophilic due to the presence of abundant carboxyl and hydroxyl groups on its molecular chain [10], which leads to poor compatibility with hydrophobic drug molecules including ibuprofen, tanshinone IIA, ciprofloxacin, triclosan, etc. [11,12,13,14]. Hence, it is difficult to achieve the effective entrapment and controlled release of hydrophobic medicines with the raw alginate and its hydrogels. In addition, the hydrophilic alginate has unpredictable and uncontrollable degradation kinetics and extensive water uptake properties, resulting in its poor stability in biological buffers, which significantly limits its practical application as a hydrophobic pharmaceutical carrier [15,16,17]. It has been reported that the broad diversity of structures and the presence of numerous functional groups available for chemical modifications represent an enormous advantage for the development of safe, non-toxic, and cost-effective micellar drug delivery systems [18]. On account of the abundant carboxyl and hydroxyl groups on the alginate’s molecular chain, the grafting of hydrophobic groups onto the alginate’s molecular chain via the chemical method may be an effective method to improve its properties such as molecular flexibility, hydrophobicity, and physicochemical and biological characteristics [10]. Thus, the resultant alginate derivative grafted with hydrophobic side groups seems to be an amphipathic polymeric surfactant which is able to realize the loading of hydrophobic drugs through its self-assembly behavior [19].

Due to the multi-functional groups in alginate, a few research strategies or methods involving the amidation, esterification, oxidation, reductive amination, and Ugi reaction for the chemical modification of alginate have been reported. Generally, these chemical modifications are mostly performed at the position of the two adjacent -OH groups (C-2 and C-3) or the position of the one -COOH group (C-6) in alginate [20]. Since there are a number of carboxyl and hydroxyl groups on the alginate’s molecular chain, these functional groups are very susceptible to the formation of the intramolecular hydrogen bonds within the molecules, which gives rise to the fully stretched rigid skeleton structure of alginate chains and decreases the reactivity of the hydroxyl and carboxyl functional groups [3,21]. Consequently, most chemical modifications require coupling agents and catalysts to activate the hydroxyl or carboxyl groups to improve the reactivity. For example, the amidation of alginate can be conducted with 1-ethyl-3-(3-dimethyl-aminopropyl) carbodiimide hydrochloride (EDC·HCl) or 2-chloro-1-methylpyridinium iodide (CMPI) as the coupling agent [17,22], and the esterification of alginate can be performed with N, N’-dicyclohexylcarbodiimide (DCC) as the catalyst [23]. Although the above modification reactions are activated with the aid of the coupling agent and catalyst, the degree of substitution of their alginate derivatives is relatively low, indicating that the reactivity is still limited. These results are presumably the result of the presence of intramolecular hydrogen bonds and the corresponding rigid structure of the alginate chains that results in the low activity of its functional groups. It is worth noting that the two adjacent -OH groups (C-2 and C-3) of the uronic acid unit of alginate can be oxidized to form multiple functional aldehyde groups with the periodate as the oxidant [24,25]. The oxidation of periodate not only opens the uronic acid of the alginate molecular chain, greatly destroying its rigid structure, but it also improves the subsequent reductive amination reaction activity of the oxidized alginate, effectively improving the molecular flexibility of alginate derivative [1,6,26]. Given the fact that the rigid structure of the alginate chains is not conducive to the self-assembly of the alginate derivative, the oxidation-reductive amination reaction that can destroy alginate’s intramolecular hydrogen bonds and rigid structure is probably the best option to endow alginate with the specific colloidal interface activity for application in pharmaceutical formulations.

In spite of the good merits of the oxidation-reductive amination reaction, the research on the oxidation-reductive amination reaction for the chemical modification of alginate is not sufficient. Kang et al. [26,27] performed the oxidation-reductive amination reaction to synthesize a series of alginate-derived polymeric surfactants with linear alkyl groups. The synthesized alginate-derived polymeric surfactants presented colloidally stable self-aggregates with a unimodal size distribution. The micelles of the alginate-derived polymeric surfactants possessed properties such as surface activity that were similar to monomeric micelles. Li et al. [28] used the alginate derivative synthesized by the oxidation-reductive amination reaction to fabricate the ibuprofen-loaded hydrogel microspheres through the cross-linking of calcium ions. The prepared drug-loaded microspheres revealed a controlled-release performance in vitro. Although these works realized the oxidation-reductive amination reaction for alginate, the systematic characterization of the alginate derivative, especially using the fluorescent pyrene probe technique and rheological method, has not been reported so far. For this case, the objective of our work was to systematically characterize the alginate derivative that is synthesized via the oxidation-reductive amination reaction, with the aim of developing pharmaceutical formulations. The synthesis of the reductive amination of oxidized alginate derivative (RAOA) through the oxidation-reductive amination reaction was carried out by using the alkyl amine as the hydrophobic modifier and NaCNBH_3_ as the reducing agent. The structure and self-assembly behavior of the resultant RAOA was systematically evaluated by multiple testing methods, including fluorescence spectrophotometry, rheology, and dynamic light scattering (DLS). Furthermore, the hydrophobic anti-inflammatory drug ibuprofen was chosen as the model drug to detect the drug loading and the release performance of RAOA. At the same time, its cytotoxicity against the murine macrophage RAW264.7 cell was also investigated. 

## 2. Results and Discussion

### 2.1. Synthesis and Characterization of RAOA

**Scheme 1:**
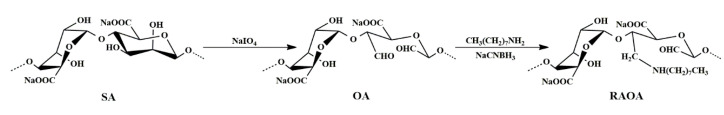
The synthesis route of RAOA via the reductive amination of oxidized alginate.

The oxidation-reduction amination reaction for the modification of SA takes advantage of the oxidizing properties of sodium periodate to oxidize the inert dihydroxy groups on the alginate uronic acid monomer to form the active dialdehyde groups [6]. As shown in Scheme 1, the ring opening of the alginate uronic acid monomer is realized in this process, which effectively destroys the rigid structure of the alginate molecular chain and improves its molecular self-assembly performance. Furthermore, the RAOA is synthesized through the primary amine condensation of alkyl amine and reduction with NaBH_3_CN. The results of the elemental analysis for the raw SA and RAOA are presented as follows. SA: C 31.15, N 0.06, H 5.56, S 0.16; RAOA: C 35.12, N 2.19, H 6.25, S 0.36. According to the change in the N/C value [3], the degree of substitution (DS) of RAOA can be calculated as 0.28 with the aid of Equation (1). 

**Figure 1:**
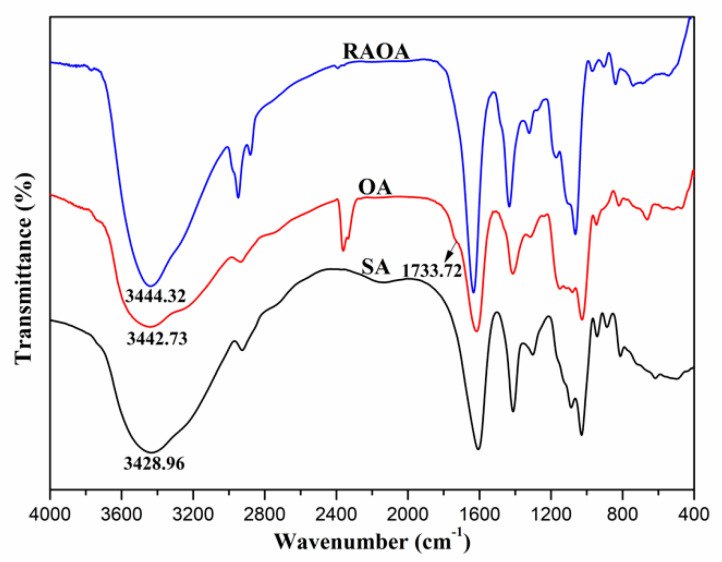
FT-IR spectra spectra of SA, OA and RAOA.

FT-IR and ^1^H NMR spectroscopy are common methods used to detect the polysaccharide molecular structure and the specific functional groups of the obtained OA and RAOA. It can be seen from Figure 1 that the strong and broad bands appearing between 4000 and 3000 cm^−1^ were caused by the hydroxyl stretching vibration of the polymers [29]. The main characteristic bands of raw SA at 2929.49, 1610.35, and 1415.56 cm^−1^ were, respectively, assigned to the C-H stretching vibration of the polysaccharide structure, and the asymmetric and symmetric stretching vibration of -COO^−^ [22]. The absorption peaks at 1093.50, 1033.71, and 949.50 cm^−1^ were attributed to the C-O and C-O-C stretching vibration on the polysaccharide skeleton, respectively [30]. In comparison with SA, the difference in the chemical structure of OA shown in its FT-IR spectrum was due to the oxidation with sodium periodate, and new weak peaks at 1733.72 cm^−1^ owing to the aldehyde symmetric vibration were observed [31]. This peak was too weak to be detected for the hemiacetal formation of the free aldehyde groups [27]. In addition, the hydroxyl stretching vibration absorption peak at 3428.96 cm^−1^ in the spectrum of SA became blue-shifted to 3442.73 cm^−1^ in the spectrum of OA, indicating that the oxidation of SA destroyed its intramolecular hydrogen bonds [30,32]. After the reductive amination of OA, the synthetic RAOA basically exhibited the same characteristic absorption peaks as SA, but the hydroxyl stretching vibration absorption peak at 3444.32 cm^−1^ became narrow and blue-shifted, indicating that the intramolecular hydrogen bond of the raw SA was destroyed in the chemical modification process [30]. At the same time, compared to SA, RAOA displayed new absorption peaks at 2950.66 and 1176.44 cm^−1^, which were attributed to the stretching vibration of -CH_3_ on the octyl group and the stretching vibration of C-O on the aldehyde group, respectively. The above results show that the hydrophobic octylamine side group was successfully grafted to the alginate backbone through the oxidation-reduction amination reaction, thus destroying the intramolecular hydrogen bond of the raw SA [33,34].

**Figure 2:**
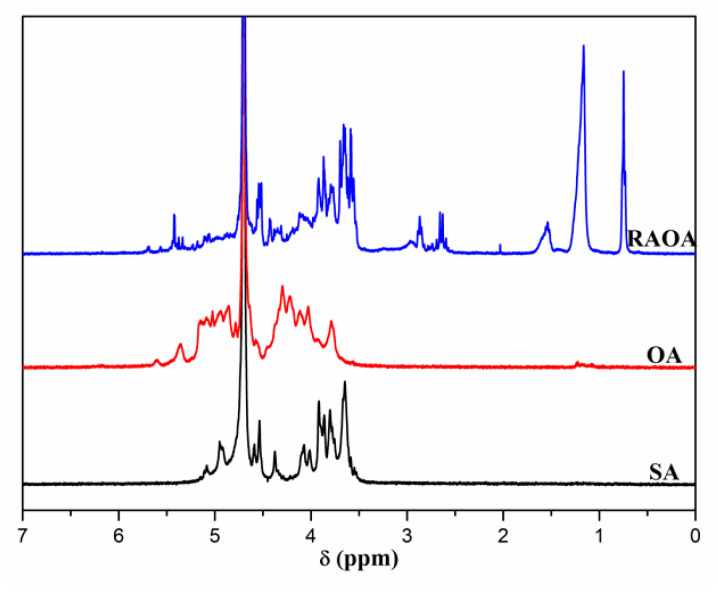
^1^H NMR spectra of SA, OA and RAOA.

Figure 2 displays the ^1^H NMR spectra of SA, OA, and RAOA. The signal peak appearing between the chemical shift (δ) of 5.5 and 3.0 ppm was ascribed to the proton signal peaks on the alginate backbone of SA [22]. In comparison with SA, the OA showed variations in the ^1^H NMR spectrum with the appearance of the new signal peaks at about 5.35, 5.02, and 4.30 ppm, which were attributed to a hemiacetalic proton generated from the hydroxyl groups of aldehyde and its neighbors, confirming the achievement of the oxidation with sodium periodate [6]. Compared with the ^1^H NMR spectrum of the raw SA, the new signal peaks of RAOA between 3.0~2.0 and 2.0~0.5 ppm were attributed to the protons of -NH-, -CH_2_-, and -CH_3_ of the grafted octyl groups, respectively [35]. The appearance of these new proton signal peaks also directly proved that the hydrophobic octylamine was successfully grafted onto the alginate backbone through the oxidation-reductive amination reaction.

**Figure 3:**
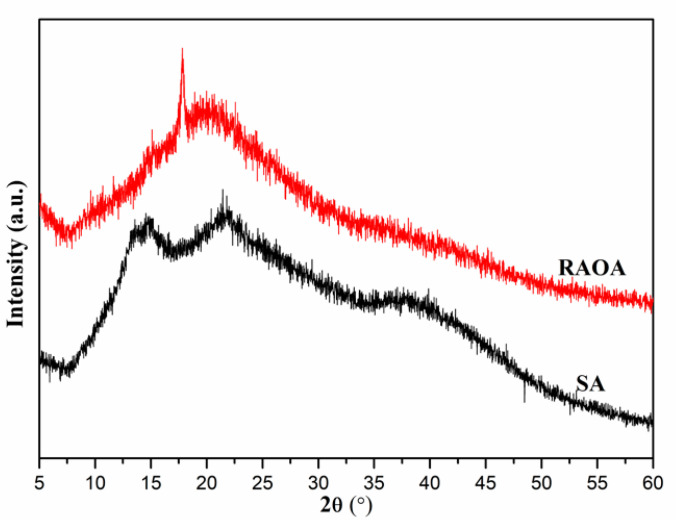
X-ray diffractograms of SA and RAOA.

XRD is the most direct and effective method to analyze the change in the crystal structure of alginate in the oxidation-reduction amination reaction for the modification of SA. The XRD patterns of SA and RAOA are compared in Figure 3. Both SA and RAOA presented weak crystalline diffraction peaks, indicating their amorphous structure. The X-ray pattern of SA showed diffraction peaks at 2θ = 14.8° and 22.3°, showing that the hydrated crystalline structure’s characteristic peaks resulted from the intramolecular hydrogen bonds of SA [36,37]. By contrast, the XRD pattern of RAOA only revealed a single broad peak at 20.1°, showing that the change in the crystal structure of SA after the chemical modification and the incorporation of the new chemical groups to the alginate backbone may cause the disruption of the regular crystallites of alginate units [3]. This result implies that the oxidation-reduction amination reaction for the modification of SA destroyed its intramolecular hydrogen bonds, which is consistent with the above FT-IR analysis.

**Figure 4:**
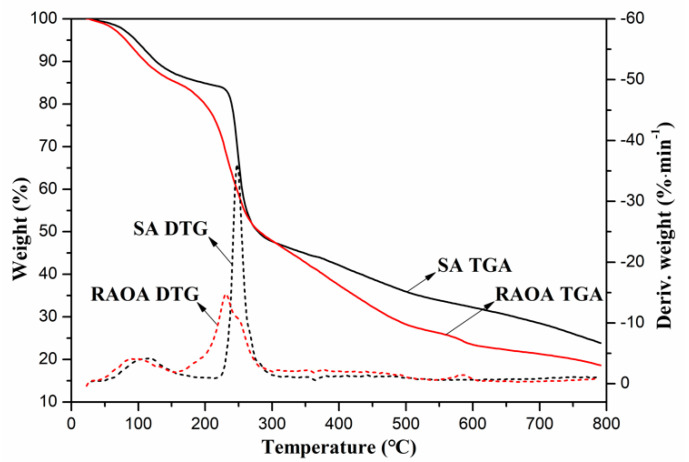
Thermograms of SA and RAOA.

The TGA measurement was utilized to estimate the different thermal stabilities of SA and RAOA. The TGA and differential thermogravimetric analysis (DTG) thermograms of SA and RAOA are shown in Figure 4. Both SA and RAOA displayed similar thermal degradation behaviors, displaying two main weight loss stages. The first weight loss stage that occurred at 80~120 °C was due to the evaporation of the physically adsorbed water from the samples [38,39], while the second weight loss stage that occurred at 200~300 °C was attributed to the thermal decomposition of the polymer materials, which were gradually thermally cracked into CO, CO_2_, and H_2_O, resulting in a rapid decline in their weight [40]. It can be also observed that the initiating decomposition temperature of RAOA in the second stage was obviously lower than that of SA, indicating that the RAOA had a lower thermal stability [34]. This result may be due to the destruction of the intramolecular hydrogen bonds of SA because of the conjugation of hydrophobic groups onto its molecular chains during the chemical modification, thereby decreasing the thermal stability of RAOA.

### 2.2. Self-Assembly Behavior of RAOA

**Figure 5:**
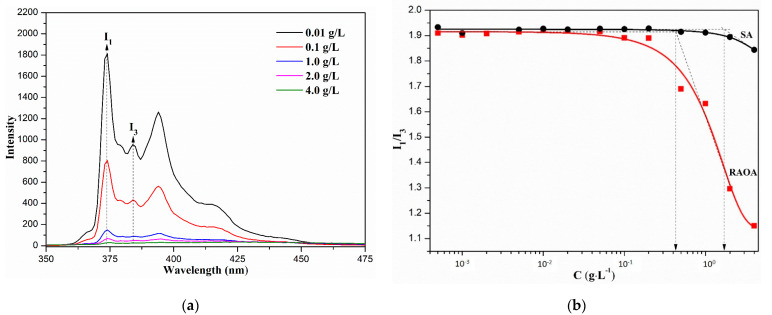
(**a**) Pyrene fluorescence spectra of RAOA in aqueous NaCl solution with various concentrations; (**b**) Plots of pyrene fluorescence intensity I_1_/I_3_ vs. the concentration of SA and RAOA solutions.

The self-assembly behavior of RAOA in NaCl solution after the oxidation-reduction amination reaction was characterized by pyrene fluorescence probe technology, which is the most effective method to verify the hydrophilic and lipophilic properties of RAOA and its critical aggregation concentration (CAC) value [41,42]. As shown in Figure 5a, the RAOA fluorescence emission spectrum of the pyrene probe revealed five characteristic peaks. The ratio of the intensity of the first emission peak at 373 nm to the third emission peak at 384 nm (I_1_/I_3_) corresponded to the micro-environmental polarity surrounding the pyrene molecules and decreased with the decline in its environmental polarity [23]. Figure 5b shows the relationship between the pyrene fluorescence intensity I_1_/I_3_ and the SA and RAOA concentrations. It can be observed that when the concentration of RAOA was low and the self-assembled micelles had not yet formed, the pyrene probe was free in the solvent, and the I_1_/I_3_ value was at a plateau. As the concentration of RAOA gradually increased, it successively formed the micellar structure with a hydrophobic cavity under the hydrophobic association. At this time, the hydrophobic pyrene probe could be solubilized in the non-polar hydrophobic cavity so that I_1_/I_3_ gradually decreased. The decrease in the ratio of I_1_/I_3_ makes clear the formation of the hydrophobic microdomain due to the intermolecular hydrophobic associations after the formation of the self-assembled micelle. From Figure 5b, the CAC value of the RAOA self-assembled micelle can be obtained from the tangent to the curve at the inflection with the horizontal tangent through the points at the low polymer concentration [3,34]. It can be seen that the CACs of RAOA and SA were 0.43 g/L and 1.65 g/L, respectively. By contrast, it can also be seen that the CAC of RAOA was significantly reduced after the chemical modification, indicating good amphiphilic properties and an enhanced molecular flexibility.

**Figure 6:**
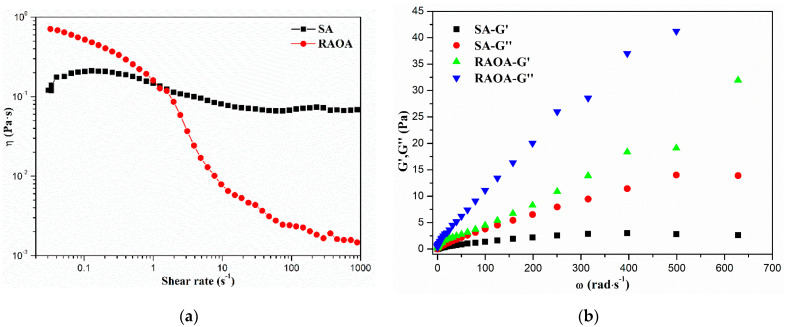
(**a**) The viscosity of the SA and RAOA aqueous solutions as a function of the shear rate at 30 °C; (**b**) Storage (G′) and loss (G″) moduli of SA and RAOA aqueous solutions as functions of angular frequency (ω). The concentrations of SA and RAOA aqueous solutions were fixed, respectively, at 1 % (*w*/*v*).

In order to further explore the effect of the oxidation-reduction amination reaction modification on the molecular flexibility of RAOA, the rheometer test was performed to investigate the relationship between the viscosity (η) of the RAOA aqueous solution and the shear rate in the steady shear measurement, the relationship between the storage moduli (G′) and the loss moduli (G″) of the RAOA aqueous solution, and the angular frequency (ω) in the oscillation frequency sweep measurement. It can be seen from Figure 6a that the viscosity (η) of the SA aqueous solution nearly displayed Newtonian liquid characteristics with the shear rate for the highly stretched rigid molecular structure of SA. However, the RAOA aqueous solution exhibited shear-thinning behavior instead [43]. This result may be due to the destruction of the rigid molecular structure of raw SA and the enhanced molecular flexibility after the oxidation-reduction amination reaction modification [21]. Moreover, RAOA formed effective chain entanglement points through self-assembly under the drive of the grafted hydrophobic groups, thereby revealing shear-thinning behavior [34]. Furthermore, it can be seen from Figure 6b that the storage moduli (G′) and the loss moduli (G″) of the RAOA aqueous solution were both higher than that of the SA aqueous solution at the same angular frequency (ω), which shows that the RAOA had probably undergone a conformational transformation driven by its hydrophobic side chains to form the effective molecular chain entanglement points, thus presenting the characteristics of a physical gel system [44]. The above rheological test results reveal that the oxidation-reduction amination reaction for the modification of SA can effectively improve its molecular flexibility to achieve the self-assembly performance.

**Figure 7:**
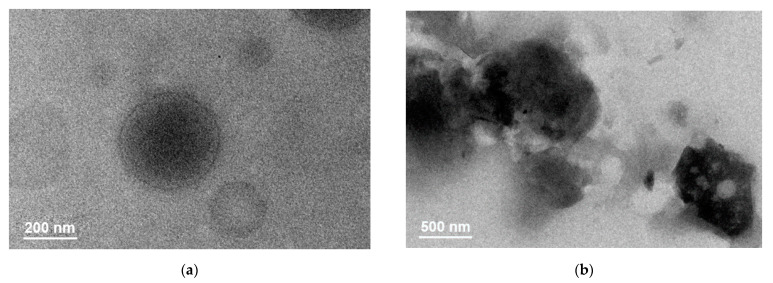

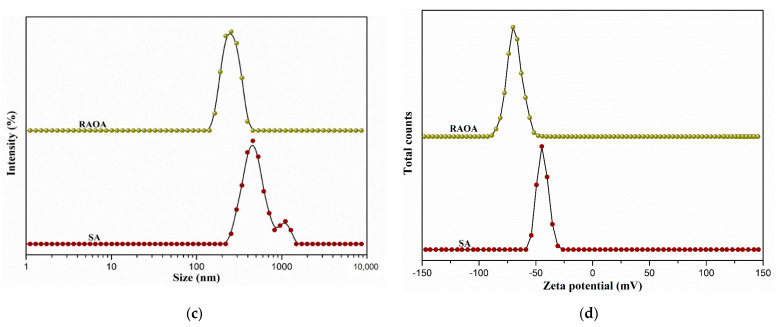
TEM image of (**a**) RAOA self-assembled micelles and (**b**) SA self-aggregates; (**c**) hydrodynamic diameter distribution and (**d**) zeta potential distribution of RAOA self-assembled micelles and SA self-aggregates.

The morphology, size, and zeta potential of the formed RAOA self-assembled micelles in the NaCl solution were further evaluated by TEM and DLS. It can be observed from Figure 7a,b that the RAOA formed regular self-assembled micelles with a spherical shape and good structural integrity through molecular self-assembly driven by the hydrophobic associations, while the SA only produced irregular self-aggregates for its rigid molecular structure resulting from the strong intramolecular hydrogen bonds [23]. This result can be ascribed to the hydrophobic associations and hydrogen bonding of RAOA that were advantageous in terms of the self-assembly behavior of amphiphilic RAOA, which not only facilitated the stability of the self-assembled micelle but also influenced the size and morphology of the micelles [45,46]. Moreover, it can be calculated from the TEM images that the average size of dry RAOA self-assembled micelles was 221 nm through the 1.2 Nano Measurer statistical software.

Additionally, the size of the RAOA self-assembled micelles was also measured by DLS, the results of which are presented in Figure 7c. It can be observed that the RAOA self-assembled micelles displayed a narrow hydrodynamic diameter distribution in comparison with the SA self-aggregates. Moreover, the average hydrodynamic diameters of the RAOA self-assembled micelles and the SA self-aggregates were 277 and 498 nm, and their PDI values were 0.19 and 0.33, respectively. The average hydrodynamic diameter of the RAOA self-assembled micelles was apparently higher than that observed from the TEM images in Figure 7a. The difference resulted from the fact that the hydrodynamic diameter of the RAOA self-assembled micelles in aqueous media is characterized the sum of the actual particle size and the thickness of water shell formed by the interactions of the hydrophilic segments, which are generally higher than the average particle size in the dry state [47]. 

In addition, it can be observed from Figure 7d that the RAOA self-assembled micelles and the SA self-aggregates exhibited dramatically negative zeta potentials at about −69.8 mV and −44.7 mV, respectively, for the presence of the negatively charged—COO^−^ [3,38]. By contrast, the absolute zeta potential of the RAOA self-assembled micelles was significantly higher than that of the SA self-aggregates, which was attributed to the hydrophilic and lipophilic properties of RAOA as well as the hydrogen bonding of the hydrophilic segments that were able to self-assemble to form the core-shell architectures where the hydrophilic carboxyl groups were exposed, thus enhancing its absolute zeta potential. It is generally accepted that the self-assembled micelles are stable in aqueous solution when the absolute value of their zeta potential is higher than 30 mV [48]. Therefore, the RAOA self-assembled micelles could be significantly stable in aqueous solution due to the strong electrostatic repulsion, thus indicating good colloidal interface activity, which could be suitable for the application in the pharmacology field.

### 2.3. Fabrication of the Drug-Loaded RAOA Microcapsules

**Figure 8:**
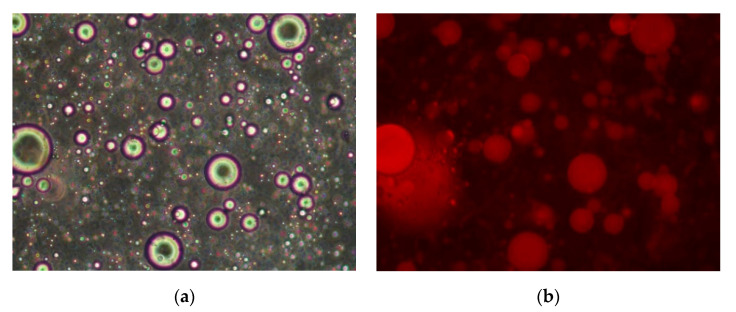
Fluorescent images of the dyed emulsions stabilized by RAOA at a final concentration of 5.0 mg/mL under (**a**) bright field and (**b**) dark field.

**Figure 9:**
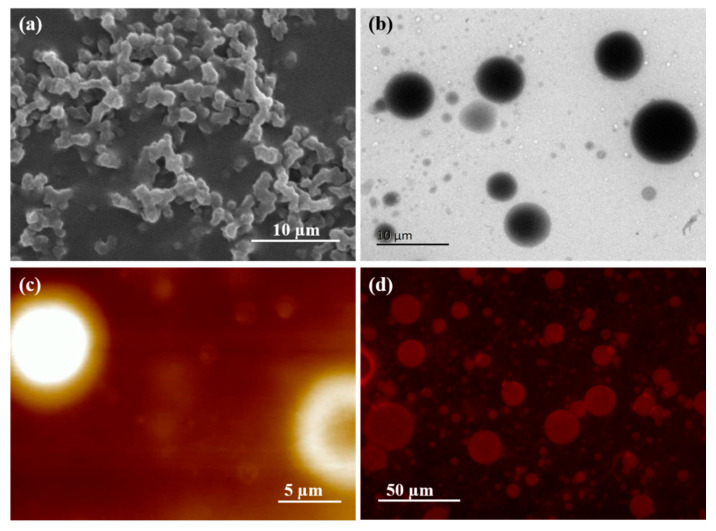
(**a**) SEM, (**b**) TEM, (**c**) AFM and (**d**) Fluorescent images of the drug-loaded RAOA microcapsules.

On account of good self-assembly performance and colloidal interface activity of RAOA, it could be used to prepare the drug-loaded microcapsules by the emulsification method. The microstructures of the fabricated drug-loaded RAOA microcapsules were observed by a fluorescent microscope after fluorescence staining, the results of which are presented in Figure 8. It can be observed that the emulsion droplets had a regular spherical shape, indicating that RAOA has emulsifying properties similar to traditional surfactants. Moreover, the emulsion droplets appeared red in the dark field for the Nile red excitation, which indicates that the drug-loaded RAOA microcapsule could achieve the effective encapsulation of chloroform with the ibuprofen because the oil-soluble Nile red only exists in the oil phase and appears red during its excitation [49]. To further observe the surface morphology and the core-shell architectures of the drug-loaded RAOA microcapsules in the dry state, SEM, TEM, and AFM measurements were also performed. The results are shown in Figure 9. Compared with the fluorescent images (Figure 9d), the SEM image of the drug-loaded RAOA microcapsules (Figure 9a) revealed a spherical shell with a shrunken and collapsed morphology due to the solvent evaporation. However, the TEM and AFM images of the drug-loaded RAOA microcapsules displayed regular spherical architectures as seen in Figure 9b,c, which were also ascribed to the good self-assembly performance and colloidal interface activity of RAOA that could be applied to fabricate the ideal hydrophobic drug carrier. In addition, drug-loaded SA aggregates were also prepared as a reference., whose EE was only 51.7% for its hydrophilic property due to the presence of large amounts of free -OH and -COOH along its backbone [10,11]. Nevertheless, the drug-loaded RAOA microcapsules had a relatively high EE of 87.6% in comparison to the drug-loaded SA aggregates (Table 1), indicating the good affinity of RAOA to hydrophobic ibuprofen, which is beneficial to the loading and controlled release of hydrophobic drug for the preparation of pharmaceutical formulations.

### 2.4. In Vitro Drug Release and Cytocompatibility of RAOA Microcapsules

The simulated release of ibuprofen from the RAOA microcapsules was realized through the swelling and degradation of the microcapsules as well as the drug self-diffusion [34,38].

**Figure 10:**
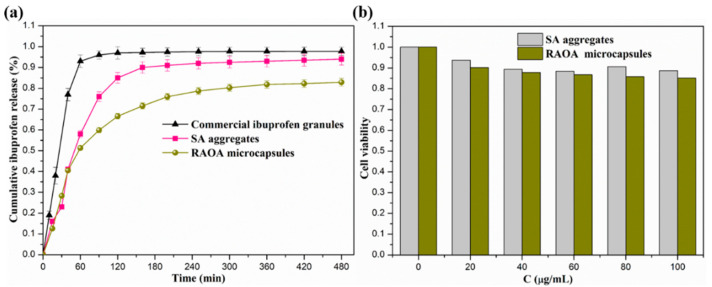
(**a**) CCK-8 assay for the cytotoxicity of RAW 264.7 cells cultured on the SA aggregates and the RAOA microcapsules after 2 days of incubation. (**b**) Release of ibuprofen from the commercial ibuprofen granules, SA aggregates, and RAOA microcapsules.

The release profiles of ibuprofen from commercially available ibuprofen granules, SA aggregates, and RAOA microcapsules are shown in Figure 10. It can be seen that the commercial ibuprofen granule released up to 90% of the total drug amount within 60 min, which is not conducive to drug absorption, and is likely to cause problems such as large fluctuations in the blood concentration and a short half-life. Therefore, in order to improve the efficacy of ibuprofen, the development of a controlled-release formulation of ibuprofen is useful. In the controlled-release formulation, the SA aggregates as the reference revealed a significant burst release and about 75% of the drug was quickly released within the first 90 min. However, the release rate of ibuprofen for the RAOA microcapsules was significantly lower than that of the SA aggregates for the same period of time, and the ibuprofen was released continuously within 300 min, indicating that the hydrophobic inner cavity of RAOA microcapsules can effectively solubilize hydrophobic ibuprofen, thus slowing down the diffusion rate of the drug and reducing the drug release rate. At the same time, the cumulative release rates of the SA aggregates and the RAOA microcapsules were 94% and 83%, respectively, which were related to the difference in EE and the affinity to hydrophobic ibuprofen between them. Moreover, it is common knowledge that a sustained release may be helpful in enhancing drug absorption in actual drug therapy [50,51,52]. The release mechanism of hydrophobic ibuprofen can be calculated with the Korsmeyer–Peppas model, which is appropriate for a cumulative release rate within 60% [53].

(1) $$M_{t}/M_{\infty }=Kt^{n}$$

where *M_t_*/*M*_∞_ is the fractional release of drug in time *t*, *K* is a constant incorporating the structural and geometrical characteristics of the delivery system, and *n* is the diffusion exponent characteristic of the release mechanism. From Table 1, it can be seen that the model correlation coefficient R^2^ of each sample was higher than 0.9, indicating that the release curves could be well fitted by the Korsmeyer–Peppas model. The characteristic diffusion exponents n, of the drug release process of the SA aggregates and the RAOA microcapsules were, respectively, 0.9241 and 0.7876 in a range from 0.5 to 1.0, indicating that their release mechanisms were confirmed as non-Fickian diffusion [54]. This result showed that the swelling and degradation of the RAOA microcapsules and the diffusion of the loaded drug jointly controlled the release rate of ibuprofen [38].

In addition, the in vitro cytocompatibility of the RAOA microcapsules was evaluated on the murine macrophage RAW264.7 cell by CCK-8 assay. It can be seen from Figure 10b that the cell viability of the murine macrophage RAW264.7 cell remained above 85% after 2 days of incubation with the concentration of the RAOA microcapsules ranging from 20 μg/mL to 100 μg/mL. Moreover, the RAOA microcapsules displayed a similar cell viability to the SA aggregates, which was close to the control. These results imply that both the SA aggregates and the RAOA microcapsules exhibited a good proliferation for the good biocompatibility of the alginate, and the RAOA had no significant cytotoxicity after the oxidation-reduction amination reaction modification [55]. Therefore, the RAOA with good amphiphilic properties, self-assembly performance, colloidal interface activity, drug loading capacity, sustained-release performance and cytocompatibility is expected to become the ideal candidate for hydrophobic drug delivery in the biomedical field.

## 3. Experimental Procedure

### 3.1. Materials

Sodium alginate (SA, M_W_ = 198,000, Mn = 136,000, G/M = 1.5) was purchased from Seaweed Mingyue Co., Ltd., Qingdao, China. Sodium periodate (98%), ethylene glycol (99.5 %), sodium cyanoborohydride (NaBH_3_CN, 95%), octylamine (99%), and model drug ibuprofen (98 %) were purchased from Aladdin Chemical Reagent Co., Ltd., Shanghai, China. Pyrene as a fluorescence probe and Nile red were obtained from Sigma-Aldrich, New York, USA. RAW 264.7 Cells were purchased from the Cell Bank of the Chinese Academy of Sciences, Shanghai, China. The DMEM medium was obtained from Gibco, Thermo Fisher Scientific, US. Fetal bovine serum (FBS) was supplied by Biological Industries, Israel. The Cell Counting Kit-8 (CCK-8) was obtained from Dojindo Chemical Laboratories, Kumamoto, Japan. Other solvents, such as anhydrous ethanol, hydrochloric acid, sodium chloride, sodium hydroxide, and phosphate buffer saline (PBS) were also purchased from Guangzhou Chemical Reagent Co., Ltd., Guangzhou, China. They were of analytical grade and were used without further purification.

### 3.2. Methods

#### 3.2.1. Synthesis of RAOA by the Oxidation-Reductive Amination Reaction

As shown in Scheme 1, the synthesis route of RAOA via the oxidation-reductive amination reaction involved the oxidation reaction on sodium alginate (SA) and the further reductive amination of the formed oxidized alginate (OA) according to the previous method with some modifications [6,27,29].

The oxidation reaction on SA was achieved with the presence of sodium periodate at an ambient temperature for a period of 24 h. The molar ratio of sodium periodate to the uronic acid monomers of SA was fixed at 30%. In detail, 2.5 g of SA was dissolved in 100 mL of deionized water to achieve 2.5% (*w*/*v*) SA aqueous solution, and 25 mL of anhydrous ethanol was added and fully dispersed in 100 mL of SA aqueous solution in a dark bottle. Then, 0.86 g of sodium periodate was incorporated into the above aqueous solution to initiate the oxidation reaction at an ambient temperature in the dark to obtain OA. After 24 h, 5 mL of ethylene glycol was added to quench the reaction under stirring for another 2 h. The resultant OA was precipitated by the introduction of 400 mL of anhydrous ethanol and 1.50 g of NaCl. After repeating the precipitation three times, the final product was dialyzed against deionized water for 5 days with the dialysis bag (molecular weight cutoff of 3500), and further lyophilized to obtain the dried OA.

Subsequently, the reductive amination of OA was carried out with alkyl amine in the presence of NaBH_3_CN. The reaction molar ratio of octylamine to the uronic acid monomers of OA was fixed at 5:1. A total of 1.47 g of octylamine was added dropwise into 100 mL of 1.5% (*w*/*v*) OA aqueous solution with vigorous stirring at an ambient temperature. After reacting for 1 h, 0.71 g of NaBH_3_CN was supplemented to perform the reductive amination reaction. A total of 24 h later, the resultant reaction solution was dialyzed against deionized water for 7 days with the dialysis bag (molecular weight cutoff of 3500), and further lyophilized to obtain the dried RAOA.

#### 3.2.2. Molecular Structure Characterization

The elementary analysis of the raw SA and RAOA was performed with a Vario EL cube elemental analyser, which could be used to calculate the degree of substitution (DS) of RAOA and the average numbers of hydrophobic chains covalently linked to hexuronic acid residues, according to the following equation:

(2) $$\mathrm{DS}=\frac{6\alpha \times M_{C}}{2M_{N}-16\alpha \times M_{C}}\times 100\%$$

where *α*, *M_C_*, and *M_N_*, respectively, represent the N/C mass ratio, the relative atomic weight of *C*, and the relative atomic weight of *N* [3].

The molecular structure of RAOA was characterized by the Fourier transform infrared spectrophotometer (FT-IR), ^1^H nuclear magnetic resonance (^1^H NMR), X-ray diffraction (XRD), and thermal gravimetric analysis (TGA). The functional groups of RAOA were characterized by the FT-IR (Nicolet-6700 FT-IR, Thermo Scientific, Waltham, MA, USA). Before the measurement, a small amount of the dried sample and KBr were mixed, ground, and compressed into disks for the test. The ^1^H NMR spectroscopy of RAOA was recorded on a nuclear magnetic resonance spectrometer (Bruker AV 400 NMR, Newark, DE, USA) at 25 °C. D_2_O (99%) was used as the solvent to prepare the sample solution in a 5 mm NMR tube with a concentration of about 10 mg/mL. The crystalline structure of RAOA was examined using an X-ray diffractometer (Bruker AXS/D8 advance XRD, Cowley Road, Cambridge, UK) with Cu-Kα radiation (λ = 0.154 nm). The measurement was performed in a step scan mode at a scanning speed of 0.025 °/s over a 2θ range of 5~60°. The thermal stability of RAOA was determined by a thermogravimetric analyzer (Q600 TGA, TA Instrument, New Castle, DE, USA). The TGA test was carried out under a N_2_ atmosphere at a heating rate of 10 K/min with the test range of 30~800 °C.

#### 3.2.3. Assessment of the Self-Assembly Behavior of RAOA

The self-assembly behavior of RAOA was evaluated by fluorescence spectroscopy, rheological measurement, transmission electron microscopy (TEM), and dynamic light scattering (DLS). The fluorescence spectroscopy of RAOA was recorded using pyrene as a fluorescence probe on a fluorescence spectrophotometer (Hitachi F7000 spectrophotometer, Honshu, Japan). A total of 0.15 mol/L aqueous NaCl solution was used as the solvent to prepare the sample solution, which was beneficial to facilitate the self-assembly of the polymer samples. The rheological properties of the RAOA solution were analyzed by a steady shear test and dynamic sweep measurements using a rotational rheometer with parallel-plate geometry (DHR TA Instruments, USA) at 25 °C. Steady shear measurements were carried out to record the apparent viscosity (η) with the shear rate ranging from 0.1 to 1000 s^−1^, while the oscillation frequency sweep measurements were conducted to record the storage modulus G′ and the loss modulus G″ with the angular frequencies (ω) ranging from 0.1 to 100 rad/s, and the strain amplitude was fixed at 1%. The morphology of the self-assembled micelles was examined by a transmission electron microscope (JEM 2100 TEM, JEOL Co., Tokyo, Japan) after the sample solution was evaporated on a carbon-coated copper grid. The hydrodynamic diameter and the zeta potential of the RAOA self-assembled micelles were examined by using a dynamic light scattering meter (Malvern Nano-ZS90 Zetasizer, Worcestershire, UK) at 25 °C. The intermolecular hydrophobic associations of the amphiphilic RAOA resulted in the formation of self-assembled micelles at a concentration higher than its CAC in 0.15 mol/L aqueous NaCl solution. Therefore, the polymeric micelle-like aggregates solution was prepared at 60 °C and fixed at a concentration of 1.0 g/L to avoid multiple scattering.

#### 3.2.4. Preparation of the Drug-Loaded RAOA Microcapsules

The drug-loaded RAOA microcapsules were prepared by the high-speed shearing method using RAOA as the emulsifier with an oil/water ratio of 1:4 (*v*/*v*). A certain amount of ibuprofen was dissolved in chloroform to prepare a chloroform solution with a drug concentration of 25 mg/mL. Then, 2 mL of this chloroform solution as the oil phase and 8 mL of the 12.5 mg/mL RAOA solution as the aqueous phase were mixed well under high-speed stirring to form the drug-loaded RAOA microcapsule emulsion through molecular self-assembly driven by the hydrophobic associations [22]. The temperature during the process was kept at 4 °C to avoid the volatility of chloroform that would affect the drug concentration. The microstructure of the drug-loaded microcapsule emulsion was observed by a fluorescent microscope (Nikon Ti-S FM, Tokyo, Japan) at 25 °C. The emulsion droplets were stained with Nile red on the glass microscope slide and covered the coverslip for the observation. Meanwhile, the surface morphology and the core-shell architectures of the drug-loaded RAOA microcapsules under the dry state were further examined by a scanning electron microscope (S-3000N SEM, Hitachi, Tokyo, Japan), TEM, and atomic force microscopy (AFM, JEOL Co., Tokyo, Japan). Additionally, the unencapsulated residual ibuprofen in the aqueous phase was extracted by chloroform, which was further measured by GC-MS (HP6890/5973MSD, Palo Alto, CA, USA) to determine the amount of residual ibuprofen. Therefore, the encapsulation efficiency (EE) for the drug-loaded RAOA microcapsules was calculated using the following equation [38,39,56].

(3) $$Encapsulation\;efficiency\;(EE)=\frac{total\;ibuprofen-residual\;ibuprofen}{total\;ibuprofen}$$

#### 3.2.5. In Vitro Release Study and Cytotoxicity

In view of the good biocompatibility of alginate, the RAOA microcapsules could be used as a kind of ibuprofen injection, so the release of ibuprofen from the drug-loaded RAOA microcapsules was conducted in a 0.1 mol/L phosphate buffered saline (PBS) medium at 37 °C, in agreement with a previous report [11]. About 5 mL of the drug-loaded RAOA microcapsules solution was encapsulated into a dialysis bag whose molecular weight cut-off was 8000, and then the dialysis bag encapsulated with the drug-loaded RAOA microcapsules solution was soaked into 100 mL PBS in a beaker. At different time intervals, the dialysis bag encapsulated with the drug-loaded RAOA microcapsules solution was taken out and then soaked in fresh PBS with the same volume to diminish the effect of the saturated solutions on the release of ibuprofen. The amount of drug released within each time interval was determined by GC-MS, and each time interval for the ibuprofen concentration was tested in parallel 3 times to take the average value.

The murine macrophage RAW264.7 cells are considered to be a representative cell line for various types of macrophages routinely used in many studies. Since RAW264.7 cells have been reported to retain many of the characteristics of macrophages in vivo that have an important role in innate immune defense against microbial infections, which involve the secretion of pro-inflammatory mediators and phagocytic activities, we used RAW264.7 cells to evaluate the cytotoxicity of RAOA microcapsules for biomedical application [57,58].The RAW264.7 cells were cultured with the culture medium that was composed of 90% DMEM, 10% fetal bovine serum, 100 U/mL penicillin, and 100 μg/mL streptomycin. The RAW264.7 cells were seeded at a density of 1.0 × 10^5^ cells/100 µL in 96-well plates with the culture medium. Then, the sterilized RAOA microcapsules were added at the final concentrations of 0, 100, 200, 300, and 400 µg/mL. These 96-well plates were then transferred to an incubator containing 5% CO_2_, 95% air, and 100% relative humidity at 37 °C. After 2 d of incubation, 10 µL of the CCK-8 reagent was supplemented into each sample, and then it was placed in the incubator at 37 °C for 4 h. Its OD value was determined at a wavelength of 450 nm using an ELISA plate reader and then converted into a macrophage cell viability as follows.

Cell viability = [(As − Ab)/(Ac − Ab)] × 100%(4)

where As is the OD value of sample group, Ac is the OD value of control group, and Ab is the OD value of blank group.

## 4. Conclusions

The research goal of the present study was to systematically characterize the alginate derivative that is synthesized via the oxidation-reductive amination reaction with the aim of developing the pharmaceutical formulations. The characterization results showed that the RAOA with a DS of 28% was successfully prepared by the oxidation-reductive amination reaction, and the grafting of hydrophobic octylamine on the backbone of alginate was conducive to enhancing its molecular flexibility and colloidal interface activity. As a result, the synthesized RAOA revealed good amphiphilic properties with a CAC of 0.43 g/L in NaCl solution, which was significantly lower than that of SA, and formed regular self-assembled micelles with an average hydrodynamic diameter of 277 nm (PDI = 0.19) and a zeta potential of about −69.8 mV. Furthermore, the drug-loaded RAOA microcapsules exhibited a relatively high EE of 87.6% and good sustained-release properties in comparison to the drug-loaded SA aggregates, indicating the good affinity of RAOA to hydrophobic ibuprofen. Finally, the cytotoxicity test results showed that the RAOA microcapsules also showed a low cytotoxicity against the RAW264.7 cell, similar to the SA aggregates. On consideration of the good merits of RAOA, such as good amphiphilic properties, self-assembly performance, colloidal interface activity, drug loading capacity, sustained-release performance and cytocompatibility, it could be a suitable material for use in hydrophobic drug delivery for biomedical applications.

## Figures and Tables

**Table 1:** Encapsulation efficiency (EE) and fitting results of release data from the Korsmeyer–Peppas model.

Formulation	EE ^a^	K (min^−*n*^)	*n*	R^2^	Mechanism
SA Aggregates	51.7 ± 4.9%	0.0123	0.9241	0.9105	Non-Fickian
RAOA Microcapsules	87.6 ± 2.8%	0.0181	0.7876	0.9337	Non-Fickian

^a^ Values for EE expressed as the mean ± the standard deviation.

## Data Availability

The data presented in this study are available on request from the corresponding author.

## Sample Availability

Samples of the compounds are available from the authors.
